# Developing an mHealth program to improve HIV care continuum outcomes among young Black gay and bisexual men

**DOI:** 10.1186/s12889-024-18652-1

**Published:** 2024-05-07

**Authors:** Aaron Plant, Paul Sparks, Deborah Neffa Creech, Ta’Jalik Morgan, Jeffrey D. Klausner, Cornelis Rietmeijer, Jorge A. Montoya

**Affiliations:** 1 Sentient Research, 231 North Walnuthaven Drive, West Covina, CA 91790 USA; 2 Keck School of Medicine, University of Southern California, 1975 Zonal Avenue, Los Angeles, CA 90033 USA; 3 Rietmeijer Consulting, LLC, 533 Marion Street, Denver, CO 82018 USA

**Keywords:** mHealth, Mobile app, Program development, HIV care continuum, Black, African American, Gay and bisexual men, Human-centered design

## Abstract

**Background:**

Young Black gay and bisexual men (YBGBM) in the United States face significant disparities in HIV care outcomes. Mobile health (mHealth) interventions have shown promise with improving outcomes for YBGBM across the HIV care continuum.

**Methods:**

We developed an mHealth application using human-centered design (HCD) from 2019–2021 in collaboration with YBGBM living with HIV and with HIV service providers. Our HCD process began with six focus groups with 50 YBGBM and interviews with 12 providers. These insights were used to inform rapid prototyping, which involved iterative testing and refining of program features and content, with 31 YBGBM and 12 providers. We then collected user feedback via an online survey with 200 YBGBM nationwide and usability testing of a functional prototype with 21 YBGBM.

**Results:**

Focus groups and interviews illuminated challenges faced by YBGBM living with HIV, including coping with an HIV diagnosis, stigma, need for social support, and a dearth of suitable information sources. YBGBM desired a holistic approach that could meet the needs of those newly diagnosed as well as those who have been living with HIV for many years. Program preferences included video-based content where users could learn from peers and experts, a range of topics, a community of people living with HIV, and tools to support their health and well-being. Providers expressed enthusiasm for an mHealth program to improve HIV care outcomes and help them serve clients. Rapid prototyping resulted in a list of content topics, resources, video characteristics, community features, and mHealth tools to support adherence, retention, goal setting, and laboratory results tracking, as well as tools to help organization staff to support clients. Online survey and usability testing confirmed the feasibility, acceptability, and usability of the content, tools, and features.

**Conclusions:**

This study demonstrates the potential of a video-based mHealth program to address the unique needs of YBGBM living with HIV, offering support and comprehensive information through a user-friendly interface and videos of peers living with HIV and of experts. The HCD approach allowed for continuous improvements to the concept to maximize cultural appropriateness, utility, and potential effectiveness for both YBGBM and HIV service organizations.

## Background

Young Black gay and bisexual men (YBGBM) face substantial disparities in HIV diagnosis and care outcomes. While gay, bisexual, and other men who have sex with men (MSM) constitute approximately 3.9% of the total U.S. population [1], they accounted for a disproportionate 67% of new HIV diagnoses in 2021 [2]. Among MSM, Black MSM experience the highest rate of HIV diagnoses by race/ethnicity, with 37% of new HIV diagnoses in 2021 attributed to this group [2]. Among 13–24-year-olds, Black youth experienced a rate of HIV diagnosis 14.3 times that of their White counterparts [2]. Furthermore, youth between the ages of 13–24 made up 24% of HIV diagnoses among MSM, but 53% of new diagnoses among Black MSM [2].

Enhancing engagement in the HIV care continuum, from diagnosis to viral suppression [3], is crucial to reducing HIV morbidity, mortality, and transmission [4–7]. However, disparities persist, as Black MSM have poorer linkage to care, retention in care, and viral suppression outcomes compared to their White counterparts, even after controlling for access to care [8, 9]. Of particular concern is that, among Black MSM living with HIV, only 65% of those ages 13–24 and only 61% of those 25–34 have achieved viral suppression [10].

Multiple factors negatively impact HIV care continuum outcomes among YBGBM, including stigma related to HIV and sexual orientation [11–16]; low self-efficacy related to health management and provider communication [17, 18]; mental health issues, such as depression, anxiety, and post-traumatic stress disorder [18, 19]; substance use [17, 18, 20–22]; and insufficient social support [23, 24]. Moreover, many of these factors intersect, creating syndemics that magnify negative outcomes for YBGBM [24–29]. In light of this spectrum of challenges, it is essential to support YBGBM using holistic approaches [30, 31] that engage them in the HIV care continuum through acceptable and accessible solutions [32, 33].

Mobile health (mHealth) interventions can positively impact HIV prevention, testing, and treatment outcomes [33], including those related to HIV self-management, and have been found to be feasible and acceptable for YBGBM [32–37]. These interventions can also be very convenient and accessible, as 96% of Americans ages 18–29 own a smartphone [38], and they can be used in rural areas where in-person HIV interventions might otherwise be unavailable [39–41]. Though evidence on the effects of mHealth strategies for stigma reduction among youth is nascent [42], emerging research suggests that these approaches can help build social support and resilience among youth who face discrimination due to their race/ethnicity or sexual orientation [43, 44].

In addition to the wide array of mHealth tools and text-based information that can be provided via smartphones, these devices enable the delivery of video-based media [32]. Prior research has found that targeted HIV prevention videos for Black MSM increased understanding and recall of messages [45]. Furthermore, online personal testimonial videos of people with chronic conditions, including people living with HIV, may improve education and social support, and reduce stigma [46]. Several studies have demonstrated the effectiveness of video-based interventions, including some specifically created for young MSM living with HIV [47–51]. These interventions have demonstrated impacts on HIV testing [47], risky sexual behaviors [47, 49], HIV disclosure [47, 49], and HIV treatment initiation [51].

Studies have identified preferences for mHealth HIV prevention and care programs in general, as well as among youth and YBGBM specifically. Desired characteristics include confidentiality protections, motivational content, both video and text content, adherence and lab results tracking, an online community, resource lists, and an overall focus on holistic health rather than only on HIV [31, 52, 53]. However, few if any mHealth programs include most or all of these features [52, 53]. In this article, we detail the human-centered design (HCD) approach used to create an mHealth app, called Amp, intended to enhance HIV care continuum outcomes and to holistically support the health and well-being of YBGBM. Amp was developed in close collaboration with YBGBM living with HIV and with HIV service providers. It includes comprehensive, video-based information, a range of mHealth tools to support HIV care outcomes, resources, and a community of people living with HIV. In the subsequent sections, we discuss the impetus for this app, the methods used to generate initial insights, the transformation of these insights into testable prototypes, and the iterative refinement of the intervention concept.

## Methods

### Inspiration for AMP

The concept for Amp emerged from a series of informal conversations between our team and HIV linkage and retention staff from a large HIV service organization in Los Angeles, CA, beginning in 2015. These staff expressed a need for programs to better engage gay and bisexual men living with HIV in care, to keep them retained in care, and to help them achieve viral suppression. We had additional conversations with staff at HIV service organizations in San Francisco, CA, Los Angeles, CA, and Huntsville, AL, in 2018. Organization staff suggested a program that could function as both a stand-alone intervention for people living with HIV and something that could be integrated into their organization’s HIV testing, linkage, and retention workflow to improve HIV care continuum outcomes.

From 2016–2018, we held individual and group discussions with 16 young gay and bisexual men who were diverse in age and race/ethnicity to assess their needs with regard to HIV care and to investigate potential solutions. These conversations revealed that despite the availability of highly effective treatment, an HIV diagnosis remained devastating. Many expressed feeling isolated after their diagnosis and overwhelmed with the information they were provided to manage their condition. When discussing possible solutions with these men, we developed the initial concept of an mHealth program (e.g., mobile website or app) that could include content (e.g., personal narrative videos and articles), tools, and a community of peers to holistically support people living with HIV. In 2018, we received funding to use HCD to fully develop the concept. We decided to focus the program on YBGBM due to the HIV care continuum disparities experienced by this group.

HCD is an approach to creating programs or products that requires a deep understanding and intensive involvement of stakeholders to ensure that programs meet the needs of the intended users [54, 55]. This process has been used to create innovative and culturally appropriate programs [56] that are high in satisfaction, usability, and effectiveness [57] among intended audiences. The mixed methods approach described below follows the stages of HCD developed by the design firm IDEO [55]. This approach is highly iterative, enabling continuous improvement throughout the development process, and refinement even after the program is launched.

### Stage 1: ideation

The first stage of the HCD process was intended to build upon the initial concept and to generate a large number of ideas for the functions, features, and design characteristics of the program. We conducted focus groups with YBGBM and key informant interviews with HIV service providers in several U.S. cities to gain a better understanding of the needs of intended users and organizations that would implement the program. Interview and focus group data were analyzed for themes, which were used to develop prototype features in the subsequent stages.

### Focus groups

From March–April 2019, we conducted six in-person focus groups with a total of 50 YBGBM, ranging from 18–30 years old (see Table 1). Focus group participants were recruited through our team’s network of contacts. Groups were held at HIV service organizations or community spaces in Alabama (Huntsville) and California (San Francisco, Los Angeles, and Oakland). Participants gave written consent and received a $100 incentive for a 90-min group. We developed a semi-structured discussion guide to investigate the experiences of YBGBM living with HIV, specifically barriers and facilitators to HIV care continuum outcomes. During the discussion, we asked for feedback on The Body, an HIV website to which patients are often referred for HIV information. We showed participants two sections, the home page and the newly diagnosed section, and asked what they thought of this website, if it seemed helpful, and how it could be improved, if at all. We then showed four existing videos available online (two of young men living with HIV and two of HIV providers) to assess preferences for video content. Finally, we read a description of our initial program concept and solicited feedback both generally and on specific content topics, tools, and features. Focus groups were audio recorded and professionally transcribed.

**Table 1:** Sample characteristics for focus groups, key informant interviews, rapid prototyping, and usability testing (% n)

	**HIV Service Providers**		**Young Black Gay/Bisexual Men**		
	Informant Interviews	Rapid Prototyping	Focus Groups	Rapid Prototyping	Usability Testing
**Variable**	*n* = 12	*n* = 12	*n* = 50	*n* = 31	*n* = 21
**Age** – Mean; Range	N/A	N/A	25.2; 18–30	25.9; 20–30	25.1; 18–29
**Gender Identity**					
Male	75%	75%	100%	100%	100%
Female	25%	25%	0%	0%	0%
**Race/Ethnicity** (mutually inclusive)					
Black or African-American	66%	83%	100%	100%	100%
White or Caucasian	33%	17%	0%	0%	0%
Hispanic or Latino	25%	0%	8%	0%	0%
**City and State**^a^					
New Orleans, LA	8%	0%	0%	0%	–-
Jackson, MS	0%	42%	0%	39%	–-
Huntsville, AL	25%	33%	26%	32%	–-
Dallas, TX	25%	0%	0%	0%	–-
Los Angeles, CA	25%	25%	28%	29%	–-
Riverside, CA	17%	0%	0%	0%	–-
Oakland, CA	0%	0%	18%	0%	–-
San Francisco, CA	0%	0%	28%	0%	–-

*N/A* Not available

^a^YBGBM who participated in usability testing were from 20 different zip codes across 10 different states

## Key informant interviews

In July 2019, we conducted 12 key informant interviews in person or by telephone with HIV service providers at five different organizations in Alabama (Huntsville), Louisiana (New Orleans), Texas (Dallas), and California (Los Angeles and Riverside; see Table 1). Providers gave either written or verbal consent and received a $75 incentive for a 60-min interview. All worked at HIV service organizations that provide case management and linkage to care to YBGBM, and 10 worked at organizations that also offer HIV treatment. We interviewed providers from different levels within the organization, including executive, senior, and frontline staff. We developed a semi-structured interview guide to solicit both general and specific input on program content, tools, and features that could be useful to YBGBM living with HIV and that would help staff to better support clients along the HIV care continuum. Provider interviews were audio recorded and professionally transcribed.

## Stage 2: rapid prototyping

Rapid prototyping is a methodology used in HCD that involves the iterative testing and refining of low-fidelity prototypes to efficiently optimize program elements. YBGBM and providers were recruited for rapid prototyping through our team’s HIV service organization contacts. Rapid prototyping sessions were carried out over two days each in California (Los Angeles), Alabama (Huntsville), and Mississippi (Jackson), with a total of 31 YBGBM ages 20–30 and 12 HIV service providers (see Table 1). Sessions took place from August–September 2019. Each session was conducted in-person with individual participants by one of three study staff. Participants provided written informed consent and received a $100 incentive for a 60-min session.

During these sessions, low-fidelity hand-drawn and digital prototypes were used to gather participants’ input on: the user interface; content, tools, and resources to be included in the program; how YBGBM might want to interact in a community of peers; ways to maximize program engagement; and whether the program should include an app, mobile website, or both. At the end of each day of prototyping, responses were integrated into revised prototypes and tested again the next day.

Prior to the rapid prototyping, we produced 13 videos on eight topics based on needs expressed by YBGBM participants and providers in the ideation stage. We varied the settings (a person’s home or a film studio), speakers (YBGBM or HIV clinician), and video characteristics (e.g., subject looking directly into the camera or off camera, and heavy or light editing) in order to give participants the opportunity to provide feedback on both video content and style. Each participant was shown at least two videos, and videos were evenly divided between participants.

### Stage 3: user feedback

#### Online survey

An online survey was conducted from September–October 2019 to gather detailed feedback on the range of program features that had been developed and refined in stages 1 and 2 and to assess the generalizability of those findings. The survey instrument was designed to capture preferences for content topics, tools, features, and resources, as well as to assess the acceptability, feasibility, and usefulness of the concept among a national sample of YBGBM living with HIV, ages 18–29 (*n* = 200; see Table 2). This sample size was sufficient given the intention was for descriptive purposes rather than statistical analysis. The survey began with questions designed to screen out participants who did not meet these inclusion criteria. Survey participants were recruited through our team’s network of HIV service organization contacts and through paid advertisements on geo-spatial networking apps and social media platforms. The survey took an average of 20 min to complete. Duplicate responses (e.g., consecutive surveys from the same IP address or similar email addresses) and responses completed in under six minutes (deemed too fast given the survey length) were excluded from analysis. Participants provided consent online before taking the survey and received a $20 incentive.

**Table 2:** User feedback survey sample characteristics (*n* = 200)

Variable	Num	Percent
**Age** – Mean (SD)	24.5	(3.1)
**Sexual Orientation**		
Gay	155	77%
Bisexual	28	14%
Queer	7	4%
Something else	4	2%
Heterosexual or straight	2	1%
Decline to answer	4	2%
**Gender Identity**		
Male	195	97%
Genderqueer / Gender non-conforming	4	2%
Decline to answer	1	1%
**Race/Ethnicity** (mutually inclusive)		
Black or African-American	171	86%
Mixed race (incl. Black or African-American)	29	15%
Hispanic or Latino (non-exclusive)	23	16%
**Sexual Partners**		
Men Only	157	79%
Men and Women	43	21%
**Health Insurance**		
Has health insurance	151	76%
Does not have health insurance	49	24%
**Even Been in Care for HIV**		
Yes, I am currently in care for HIV	158	79%
I was in care for HIV, but I'm not currently	22	11%
I have never been in care for HIV	20	10%
**How Long Ago was Your HIV Diagnosis?**		
Within the last month	4	2%
Between 2 and 6 months	23	12%
Between 6 months and 1 year	32	16%
Between 1 and 2 years	47	23%
Between 2 and 5 years	54	27%
More than 5 years	35	18%
Unsure	5	2%
**Education**		
Junior high school	1	1%
Some high school	11	5%
Completed high school	39	20%
Some college/trade school	101	50%
College graduate	38	19%
Graduate work or graduate degree	8	4%
Decline to answer	2	1%
**Income Range**		
$0 to $9,999	40	20%
$10,000 to $19,999	25	13%
$20,000 to $29,999	42	21%
$30,000 to $39,999	31	16%
$40,000 to $49,999	15	8%
$50,000 to $59,999	11	5%
$60,000 to $79,999	9	4%
$80,000 to $99,999	4	2%
Decline to answer / Don’t know	23	11%

## Usability testing of the functional prototype

Using data from stages 1 and 2 and the online survey, we worked with a website developer to create a working digital prototype of the app. The objective of the online usability testing was to identify potential functionality issues and use the findings to maximize program acceptability and effectiveness. Participants were sent a link to the prototype and asked to view it on their smartphone. The prototype included the proposed topics, tools, and videos developed in stage 2, but was designed in grayscale without any branding or images. This approach allowed us to test usability of the interface without distracting users with design elements.

Usability testing participants (*n* = 21; see Table 1) were recruited through our team’s network of HIV service providers and from the online survey, with participants contacted in the order in which they completed the survey. Usability testing sessions were conducted in October 2021, with study team members observing participants interacting with the prototype over Zoom. During the session, participants were presented with several tasks related to finding information, tools, or resources on the prototype (e.g., “find and use the medication reminder” or “find a video about disclosing HIV status to sex partners”). Participants were also asked open-ended questions about topics, community features, tools, and resources; ease and predicted frequency with which they would use the app; and whether most of the content should be video- or text-based. Finally, each participant orally completed the System Usability Scale, or SUS [58], a reliable tool for measuring usability comprised of 10 questions with five response options, from “strongly agree” to “strongly disagree.” Each testing session lasted approximately one hour. Participants provided verbal consent and received a $100 incentive.

## Analyses

Stage 1 focus group and interview transcripts were coded using deductive and inductive approaches by two of the team members experienced with qualitative data analysis. They developed codes based on the interview and discussion guides and added codes as they emerged from the data. Transcripts were coded to identify themes relevant to developing the app and its components (e.g., content, tools, community features, and resources). In stage 2, written notes of participants’ feedback during rapid prototyping sessions were entered into a digital spreadsheet after each session and were categorized into prototype elements that received positive feedback and those needing improvement. In stage 3, survey data and the SUS results were assessed using descriptive statistics (e.g., frequencies) in SPSS v.26. During usability testing of the functional prototype, task completion times were captured by the interviewer using a timer and were used to calculate mean and range values. Qualitative responses were analyzed for common themes with regard to satisfaction with content, tools, usability, and likely uptake of the program.

## Results

### Stage 1: ideation

Seven main themes emerged from the focus groups and interviews. Sample quotes for each theme can be found in Table 3.

**Table 3:** Sample quotes from YBGBM participants and HIV service providers

Theme	Sample quotes
Learning about HIV after a new diagnosis is overwhelming, and available online resources do not meet the needs of YBGBM	*I think that's pretty much what people are looking for [online], looking for hope in a sense, along with information*. – YBGBM, Huntsville *I think some of the information out there is printed in such medical terms, it's not in layman's terms. You get overwhelmed trying to understand what the language even means*. – YBGBM, San Francisco *A lot of them [YBGBM clients] use WebMD, which is terrible because they diagnose themselves with several other things after they get the HIV diagnosis. And then we get the calls the next day.* – Los Angeles, Prevention youth programs training specialist
Participants expressed a desire for video-based content and programs	*I watched a lot of videos on YouTube about it [HIV] because I was diagnosed this year… Just people going through it. I felt alone, so I just wanted to hear other people’s stories and how they found out and how they got through it, the medication, insurance… I just did a lot of research.* – YBGBM, Huntsville *I like the videos, not having to read. I'm not a big reader, but that's just me personally*. – YBGBM, Huntsville *The more I see people that are going through the same thing, the more I'm going to be able to face the fact that, I'm not by myself, because I feel like that right now, but no matter what, I’ve got to keep on remembering that I'm not by myself in a situation like this.–* YBGBM, Los Angeles *Giving them a packet and saying, “Hey, read this. This is going to tell you what you need to know about HIV, adherence, and all this,” I don’t think will be very effective now, in 2019. Videos will very much be effective, because that’s the world that these young MSMs live in. Videos, and phone, and technology, and social media*. – Dallas, Case manager
YBGBM need more support related to disclosure and stigma, which can impact their HIV care outcomes	*I disclosed to my best friend first and she completely just went silent. That deterred me from actually even telling anyone. … I ended up telling my uncles. The reaction I was expecting was not what happened. They were super supportive and understanding, and super comforting, and they immediately went to help me out with making my appointments and getting everything done*. – YBGBM, Los Angeles *My grandma had me eating off of paper plates, plastic spoons and forks for a whole year. … Cousins bleaching the shower down after I get out of the shower.* – YBGBM, Los Angeles *Stigma keeps them from going to the doctor. Stigma keeps them from going to get treated….Or some people are like, “I don’t like to take my medicine on me because someone’s going to see it*.” – Dallas, Case manager *A lot of them, I’ve noticed that once they get the diagnosis, they kind of shy away from services, because they’re terrified or scared because of the stigma that is still out there. They don’t want their friends to find out. They don’t want family members to find out. They don’t want their partner to find out, because they fear being left or abandoned.* – Los Angeles, Associate director of HIV prevention services *Internalized homo-negativity and homophobia are rampant, and it’s a very sensitive topic for some people. … I think that [disclosure] presents an extra challenge in addition to misinformation around HIV, and stigma around that makes it difficult for them to interact and disclose this type of information to others around them, which most likely impacts their accessing and engagement in services.* – New Orleans, Director of recruitment, engagement, and retention
YBGBM would benefit from opportunities to learn and get support from others living with HIV	*[On a website for people living with HIV] I would include an anonymous way to talk to other people in the same situations. The reason why people get on Jack’d and get on Grindr is because, I mean it's really one of the only logical ways to meet other people in the same situation as you. Other homosexual men. … They don't have some anonymous way for people with HIV to talk with each other.* – YBGBM, Huntsville *Finding other people who are dealing with the same thing you're dealing with… that’s hope. It makes you want to fight even harder. Like, you know, I can do this, it’s not the end of the world. I can still have a family, I can still have someone, I can still be as healthy as the next person. Not let this be the end of me*. – YBGBM, Huntsville *What I would like to hear is basically that reassurance from other people living with it, that there is… it's not a death sentence. There is help, that everything's going to be okay. I think that was my biggest worry, was like okay, I definitely… what to do, I'd feel totally lost. I think that would be really good*. – YBGBM, San Francisco *It would be nice if they had… extra support from other people around their age groups so that they can share and compare and build each other up in that regard. I think it allows them to see the importance of people who are taking their meds, who are keeping up with their appointments. It gives them that motivation, it gives them someone to look to as, “Okay, if they can do this, I can do this, too.”* – Huntsville, Case manager
A holistic approach would be most useful for people living with HIV and for service organizations	*When it comes to young Black men, programs need to be designed to really treat the whole person, as opposed to just focus on getting them into care. … Once you’ve looked at the whole person, then use that as a way to get them into care, and to keep them in care*. – Los Angeles, Director of HIV prevention services *I think the holistic approach [is really usable]…So knowing that I’m clicking a website or an app that’s going to tell me how to live my best life, despite just getting diagnosed with HIV, I feel like a lot of people will gravitate to that. Once they see the uplifting message of, “Here’s how to thrive, by so and so and so,” it’ll be great. I think that that will be really effective*. – Los Angeles, Prevention youth programs training specialist *I think it would be a good resource because we don’t have a lot of stuff to refer clients to, like when they’re not within our arms’ reach, and I think that it would be a good addition to let them know that there is something out there that is concerned about their holistic self when we’re not around*. – Los Angeles, Associate director of HIV prevention services
YBGBM want an HIV program that is not exclusively focused on them	*It [the app/website] shouldn’t single out one race or gender, because then it makes it seem like we [Black gay/bisexual men] are the only ones with this issue [HIV]*. – YBGBM, Huntsville *I personally would not use anything like that, that was not open to everybody. If it was only for a specific race, I wouldn’t use it.* – YBGBM, Huntsville *It [the app/website] should be for Black people, but it should encompass all of those folks [other race/ethnicities]. *– YBGBM, Oakland *Having faces of different colors, I think that’s more beneficial*. – Los Angeles, Prevention youth programs training specialist
Digital programs can help HIV providers and their organizations reach HIV care continuum outcome goals	*I think an app like this would help… with our newly diagnosed HIV positives that need help guiding through this new way of life… “Hey, you’re newly diagnosed; let me show you something real cool.” *– Dallas, Case manager [*Having videos of organization staff] would be awesome. Anything to help [clients] feel like they are connected to us I think is important. *– Huntsville, Case manager *You risk losing them after that [first visit after diagnosis], so I think that that constant reminder of, “Once you leave here, you’re still going to have questions. You just got diagnosed, you’re still going to have questions, this app is going to be your best friend.”* – Los Angeles, Prevention youth programs training specialist *I think this is a tool that we would definitely be able to use with our clients. As you talked about, the medication reminder, the appointment reminder, the resources that are readily available, the option, once they are ready to tell their experience, strengthen the hope, I think is very important. … I see it as a complement to the work that we’re already doing*. – Dallas, Program manager

#### Learning about HIV after a new diagnosis is overwhelming, and available online resources do not meet the needs of YBGBM

Many focus group participants described being devastated after receiving their HIV diagnosis (e.g., feeling isolated, depressed, and afraid of dying and social rejection). Participants also reported having many questions about living with HIV (e.g., effects of HIV and medications on long-term health, and transmission to partners). Many participants felt overwhelmed with receiving so much information about HIV from their providers right after their diagnosis.

Participants reported seeking HIV information online, often even years after their diagnosis. Several participants stated that it was difficult to find trustworthy and accurate information. When participants were shown The Body website, many felt it was too text-heavy and visually busy to easily find needed information.

Most interviewed HIV service providers said that they wanted a medically accurate, easy-to-navigate informational website to offer clients, but that they were not aware of such a resource. Few providers reported referring clients to websites or apps, other than several who recommend apps specifically for medication adherence. Providers said that clients receive HIV information, including written materials, during the first clinic visit after their diagnosis. However, many providers said clients struggle with important concepts related to living with HIV (e.g., CD4 count and viral load) and would benefit from ongoing education.

#### Participants expressed a desire for video-based content and programs

YBGBM participants and HIV service providers felt that the new program should include videos of both people living with HIV and experts. Participants and providers said that program videos (and the content overall) should be informational and motivational, should feel authentic as opposed to overly polished, and should include a diversity of stories (e.g., people at different stages of living with HIV, and stories of challenges and successes). Some YBGBM and providers felt that the content should be delivered through both text and video, as people learn in different ways.

When participants were shown sample video testimonials of other young men living with HIV they responded very favorably; many said that seeing peers speak openly about their HIV status and sexual orientation was motivating and empowering. Some providers felt that including videos of peers navigating challenges related to living with HIV could increase YBGBM’s self-efficacy related to care continuum outcomes and help them build skills to stay healthy (e.g., videos could show YBGBM discussing their strategies for taking medication and staying in care). They also thought that the program could reduce fears and offer hope by showing people thriving while living with HIV.

#### YBGBM need more support related to disclosure and stigma, which can impact their HIV care outcomes

Numerous YBGBM participants across groups described the impact that disclosure issues and HIV stigma have had on their lives. Some YBGBM reported positive experiences with disclosure, describing telling others as cathartic and facilitating social support. However, many shared fears or negative stories of disclosing their HIV status, with some experiencing stigma from friends, partners, and family members, which added to feelings of isolation. YBGBM participants also said HIV stigma within the gay community (e.g., on dating apps) exacerbated challenges with finding partners and establishing romantic relationships. Both YBGBM and providers talked about stigma being related to fear and misinformation about HIV transmission (e.g., family members believing that HIV can be transmitted from hugging or sharing dishes). Several providers suggested that the program provide accurate information to family members to reduce this stigma and demonstrate how to support a loved one living with HIV.

YBGBM participants and providers mentioned disclosure issues and stigma as barriers to accessing HIV care, to medication adherence, and to staying in care. For instance, some YBGBM reported missing medical appointments due to fears of being seen at a clinic or missing medication doses because they did not want to take the pills in front of others. Both providers and YBGBM participants suggested that the program include videos with examples for when and how to disclose their status. Further, participants and providers emphasized that the program should address confidentiality concerns (e.g., HIV should not be in the program name).

#### YBGBM would benefit from opportunities to learn and get support from others living with HIV

Most YBGBM participants said that they rarely discuss HIV with anyone, even with others whom they know are living with HIV. However, participants in all groups discussed the value of getting support and information from other people living with HIV. Providers also emphasized the importance of spaces where people living with HIV can support one another in a safe environment. Both YBGBM and providers responded enthusiastically to the idea of building a community of people living with HIV where they can share their experiences (e.g., through creating a profile, direct messaging, or posting on a forum) as part of the program. Some providers also suggested that the program could include trained peer mentors living with HIV who can answer questions and provide support and resources.

#### A holistic approach would be most useful for people living with HIV and for service organizations

Participants across focus groups wanted a wide range of content about living with HIV, including information about medical treatment, disclosure, sex and relationships, nutrition and exercise, mental health, and resources. Participants explained that having holistic content would be most beneficial, as information needs vary from person to person and also change over time (e.g., right after a diagnosis versus several years later). All HIV providers felt the program should include content, tools, and resources on a wide range of issues that affect well-being and HIV care outcomes of YBGBM (e.g., unaddressed mental health issues, disclosure, stigma, substance use, HIV misinformation, medication adherence, awareness of available resources, and medical distrust). Providers felt that a “one stop shop” approach would make the program more acceptable, engaging, and useful to both YBGBM and to HIV organizations.

#### YBGBM want an HIV program that is not exclusively focused on them

The majority of YBGBM participants felt that the program’s videos and community should represent people living with HIV of diverse races/ethnicities, gender identities, sexual orientations, and experiences. Many participants said that they would not relate to a program that was only for Black men living with HIV, or they felt that this would be stigmatizing (as a program for only YBGBM would suggest that they are the primary group affected by HIV). Others felt that a diverse community of people living with HIV should be included yet the experiences of young people of color should be emphasized. Very few participants felt that the program should only be for YBGBM.

#### Digital programs can help HIV providers and their organizations reach HIV care continuum outcome goals

All HIV service providers interviewed felt that an mHealth program could help them to support their clients through linkage, treatment initiation, adherence, retention, and viral suppression. Providers mentioned several features that could be useful, including a messaging platform that could be used by case managers, clinic appointment and medication reminders, and a way to track CD4/viral load test results. Providers at organizations that already offer some of these features (e.g., a messaging system) thought that an mHealth program could be very useful for other components (e.g., videos addressing HIV stigma, HIV education, and a community for people living with HIV). Providers suggested that the program could be offered to clients as soon as they receive a diagnosis in order to provide social support and encourage linkage during that critical time. All providers said that their staff are open to using technology and that the proposed mHealth program could be feasibly integrated into their workflow.

### Stage 2: rapid prototyping

Insights from the focus groups and interviews were incorporated into the development of a series of prototypes covering potential content topics, sample videos, tools, and resources (See Table 4 for ways that findings from each stage of research were used to improve the program).

#### Content

When presented with a list of program topics and sub-topics, participants expressed that the proposed content was comprehensive and that it would appeal to people at different stages of living with HIV. When asked which topics would be most helpful to them personally, participants offered a range of responses, which supported findings from stage 1 that having holistic content would engage a broader audience. All participants emphasized the value of information specifically for individuals newly diagnosed with HIV and said that content should emphasize that people can lead a long, healthy life with HIV. Specific topics that participants found helpful included: U = U (Undetectable = Untransmittable); that people living with HIV can have a fulfilling love life and sex life; mental health (e.g., addressing depression and anxiety, and dealing with trauma and stress); maintaining overall health (e.g., diet and exercise, preventing sexually transmitted infections, and managing substance use); understanding medications and their side effects; and examples of how to disclose their HIV status to partners, family, and friends.

Nearly all participants supported the idea of delivering most of the content through videos featuring individuals living with HIV and experts. The majority preferred video over text-based content or desired a combination of video and text. Similar to focus group participants, prototyping participants expressed the desire for videos featuring diverse people living with HIV. Participants also liked the idea of featuring videos of doctors, mental health specialists, and case managers from the organization where they receive their HIV care. Participants suggested several ways to keep them engaged with the program, such as incorporating motivational content (e.g., daily affirmations and tips), regularly updating the program with new videos, sending notifications about new content (via app notifications or text messages), the ability to post their own content, and having a way to save and share content.

Participants responded favorably to the 13 sample videos we created and found the people in the videos to be relatable and authentic. Most considered the video lengths (ranging from 1.5 min to 4.5 min) to be appropriate. Notably, some participants who did not favor a particular video indicated they would simply switch to another video rather than discontinue using the program. We tested versions of these videos where the subject did or did not speak directly into the camera, with participants overwhelmingly preferring the former.

#### Tools

Proposed tools, including adherence and appointment reminders, viral load and CD4 trackers, a tool to meet health and personal goals, and a notepad to write questions for providers received highly favorable feedback. Some participants expressed the desire to interact with mentors or case managers through the program. Others suggested customizability or the inclusion of games. While some participants said they might not use every tool, they felt that including a range of tools was needed to meet the diverse needs of people living with HIV.

#### Community

Participants strongly advocated for having various ways to interact with other people living with HIV, including creating a profile, direct messaging, and responding to questions on a forum. They expressed a desire to share information and offer help to others, and they emphasized the potential for such a community to reduce stigma and isolation, and facilitate the exchange of useful information, strategies, and support. Some participants stressed the importance of mechanisms to address online bullying and proposed rules for community engagement, such as prohibiting trolling and having methods for removing users who breach community guidelines. One participant recommended creating online support groups centered around specific topics.

#### Resources

Most participants felt the resources proposed, which included HIV care and treatment, mental health, housing, food assistance, career, legal, and education were comprehensive and useful. Participants emphasized the need to understand the full range of available resources and to provide instructions and support for accessing them. Several participants suggested including hotlines as additional resources.

#### Rapid prototyping with providers

Providers reacted enthusiastically to the prototypes and felt that the mHealth program would help improve HIV care continuum outcomes. They felt it would provide their clients with an efficient means of engagement and support, as well as enhance the provision of medically accurate information. Some providers proposed introducing the app to newly diagnosed clients on their first visit to support linkage into care and treatment initiation. One provider noted that newly diagnosed people are often overwhelmed with information, which makes it difficult to retain. This provider felt the program would allow clients to access important information when they are ready to receive it. Providers also suggested potential improvements, such as renaming specific topics and including a feedback mechanism so clients could suggest service improvements for their organization. One provider early in stage 2 suggested developing a dashboard to enable staff to support clients as they use the app (e.g., a tool to message clients and suggest specific content or tools to assist them with specific challenges). This idea was tested with all subsequent providers and they were all very supportive of this feature. All providers considered the mHealth program to be feasible for implementation within their organization Table 4.

**Table 4:** Improvements to the program concept after each stage of research

**Improvements after stage 1**
Substantial refinements were made to the program concept using findings from the focus groups with YBGBM and interviews with HIV providers. These included additional content areas and new or improved tools and features further explored in stages 2 and 3. Improvements included: • Multiple ways to protect user confidentiality. Confidentiality was a concern among many YBGBM. We learned that simple steps could allay such concerns, such as making the program name discreet, having both an app and a website (for anyone reluctant to download an HIV app), and allowing anonymous registration • Including a wide range of content and tools to meet the needs of diverse users. The findings indicated that different people would interact in different ways with the app. We included various tools and content to improve usability and uptake • Adding videos for families. YBGBM and providers discussed stigma from family members. We added videos that YBGBM can share with family, such as a video of a medical doctor clarifying that HIV cannot be transmitted through casual contact (e.g., eating from the same dish) and videos of family members discussing how they have changed to be more supportive • Motivational content. YBGBM and providers suggested that the program could integrate motivational statements and affirmations (e.g., a daily affirmation upon opening the app), and include content that is empowering and informational (e.g., an explanation of how taking HIV medications every day can help one live a long, healthy life) • New tools. Additional tool suggestions included a pharmacy refill reminder and a notepad to store questions or to record side effects to discuss with their provider at their next visit • Peer mentors. Providers and YBGBM responded favorably to the proposed community features of the program and felt this would help address the social isolation experienced by many YBGBM living with HIV. An additional feature suggested was to have trained mentors on the app to answer questions and provide support
**Improvements after stage 2**
Numerous improvements were made to the program concept using data collected during rapid prototype sessions with YBGBM living with HIV and with HIV providers. Changes were made iteratively and were subsequently tested with the next participants. Improvements included: • Making medication reminders motivational. YBGBM and providers wanted motivational elements in the program. In the focus groups, many mentioned that adherence struggles were due to not wanting to take medications rather than forgetting to take them. We added a message in adherence reminder congratulating users for the number of days in a row that they had taken their medications • Virtual support groups. One YBGBM participant suggested that the program could include ongoing support groups for different topics (e.g., using substances, dealing with a diagnosis, and thriving with HIV). This idea tested very well with subsequent YBGBM and provider participants • Videos for partners and friends, in addition to family members. Focus group participants suggested creating videos for families to help them support (and not stigmatize) people with HIV. One YBGBM participant early in rapid prototype testing suggested including videos of how partners or friends should react when disclosed to. We tested both ideas extensively and they were well received • Tools to help users set and attain goals. One YBGBM participant suggested adding a tool to help set and reach goals, whether related to HIV directly or to other issues (e.g., eating healthy). This tested well with YBGBM and provider participants • Provider dashboard. A provider suggested creating a dashboard that organization staff could use to engage with clients as they use the program. This idea tested well with all subsequent providers and nearly all YBGBM participants
**Improvements after stage 3**
Stage 3 aimed to test the program concept from the perspective of a larger, more geographically diverse sample and to conduct user testing to identify any issues prior to building the program. In usability testing, most participants were able to complete their tasks very quickly and gave very positive feedback on tools, community features, resources, inclusion of both video and text content, and ease of use, including that the content and tools felt comprehensive (i.e., nothing was missing). Thus, stage 3 reinforced that program concept elements developed in stages 1 and 2 were feasible and accessible, rather than identifying areas for improvement. Several important program elements confirmed included: • That the program should include representation of people living with HIV who are diverse in terms of race/ethnicity, gender, gender identity, sexual orientation, and age, rather than focusing exclusively on YBGBM • An app was preferred over a mobile website (although we will add a mobile website version when funding permits)

### Stage 3: user feedback

#### Online survey

Results from the online survey among YBGBM nationwide echoed the findings from stages 1 and 2 (See Table 5). For instance, a high percentage of respondents indicated that: including topics such as mental health (92%), recent diagnosis (91%), and sex and relationships (86.5%) is “very important”; having a community of young men living with HIV to communicate and share experiences with through the program would be “very helpful” (77%); and the program’s community should not be exclusive to YBGBM living with HIV (90%).

**Table 5:** User feedback survey results (*n* = 200)

**Topics, content, and videos**	Percent of Sample (%n)		
*How difficult have each of these things been for you in the past year*?^a^	**Very difficult**	**Somewhat difficult**	**Not at all difficult**
Finding a relationship	37.5	34.5	28
Feeling isolated or alone	34.5	35	30.5
Telling friends or family about your HIV status	34.5	41.5	24
Telling partners about your HIV status	30	33	37
Feeling sad or depressed	29.5	44	26.5
Experiencing HIV stigma/discrimination from family, friends, or society	27	27.5	45.5
Taking your HIV medications (because you forget)	19.5	38	42.5
Accessing resources	17	37.5	45.5
Talking to your doctor about HIV-related issues	12	33.5	54.5
Taking your HIV medications (because you don’t feel like taking them)	10.5	31	58.5
Making it to appointments for HIV care	10	29.5	60.5
Refilling your prescriptions	9	34.5	56.5
*How important would each of these topics be to include in the app/mobile website*?^a^	**Very important**	**Somewhat important**	**Not at all important**
Mental Health (e.g., getting support, dealing with isolation, depression, managing stress)	92	8	0
Just Diagnosed (e.g., dealing with a new diagnosis, first steps, starting treatment, HIV 101)	91	8	1
Staying Healthy (e.g., medications, side effects, working with your doctor, nutrition/exercise, substance use, STIs)	89	10.5	0.5
Sex and Relationships (e.g., finding love, disclosing to partners, HIV negative partners, hookup apps)	86.5	13	0.5
Your Best Life (e.g., reaching your goals, staying active, expressing yourself, community service)	76.5	17	6.5
Telling Others (family, friends, partners, others)	74.5	21.5	4
*How important would it be to have each of these types of content*?^a^	**Very important**	**Somewhat important**	**Not at all important**
News about HIV (new treatments, efforts to find a cure)	78.5	19.5	2
Frequently asked questions	78.5	20.5	1
Articles	52	44.5	3.5
Affirmations and motivations (e.g., a daily affirmation)	47.5	32.5	20
*When it comes to the videos of people living with HIV on the app, who would you want to see included?*	**Percent**		
Anyone living with HIV, of any age, gender, or race/ethnicity	51.5	–-	–-
Young men living with HIV, of any race/ethnicity	22.5	–-	–-
Young Black/African-American men living with HIV	12.5	–-	–-
Young men of color living with HIV	7.5	–-	–-
Men of color living with HIV, of any age	4.5	–-	–-
Other	1.5	–-	–-
**Tools, resources, and other features**	**Percent of Sample (%n)**		
*Which of these tools would you use*?^a^	**Definitely would use**	**Might use**	**Definitely would not use**
CD4 and viral load tracker (to chart lab results over time)	81.5	16	2.5
Your medications (tracks your medications and dosages)	75	22	3
Appointment reminder (would link to your smartphone calendar)	62.5	29	8.5
Medication reminder (a discreet reminder that can be customized for your needs)	61.5	29	9.5
Pharmacy pick-up reminder (would link to your smartphone calendar)	56.5	32	11.5
Custom tracker (allows you to create your own tracker)	46	43.5	10.5
Provider messaging (privately communicate with your doctor or case manager if you have one)	46.4	40	13.6
Notepad (for recording questions for your provider, symptoms, side effects, and more)	41	46.5	12.5
*Which of these community features would you use*?^a^	**Definitely would use**	**Might use**	**Definitely would not use**
Ask question in a Q & A forum/message board	66	30.5	3.5
Join a virtual support group on the app	64.5	32.5	3
Direct message (DM) other members	63.5	30.5	6
Join a chat with an expert (discussing a topic like relationships, telling family members, or side effects)	62	36	2
*How helpful do you think it would be to include each of these resources?*^*a*^	**Very helpful**	**Somewhat helpful**	**Not at all helpful**
Mental health and support (including support groups)	80	19	1
Health care	77.5	20.5	2
Job and career	76.5	20.5	3
Help quitting alcohol, tobacco, or drugs	69.5	26	4.5
Housing	64	22	14
Hotlines	63	29	8
Food	61.5	23	15.5
*How helpful would it be to have a community on the app of other young men living with HIV to communicate with and to share experiences with*?	**Very helpful**	**Somewhat helpful**	**Not at all helpful**
	77	23	0
*How useful would it be to have a feature with trained mentors who can answer question on the app/website*?	**Very useful**	**Somewhat useful**	**Not at all useful**
	72	27.5	0.5
*Which of these ideas would make the app most engaging to you and keep you coming back*?^a^	**Very engaging**	**Somewhat engaging**	**Not at all engaging**
Interacting with other people living with HIV in the app community	83.5	14.5	2
Regularly adding new videos of people living with HIV telling their stories	73.5	23.5	3
Commenting on videos, content, and posts	73	24	3
Resources available through the app	73	25.5	1.5
Regularly adding new videos of experts talking about different topics related to HIV	72.5	27	0.5
Tools available through the app	65.5	31.5	3
Being able to customize your experience on the app	64	23	13
Notifications of new content and tools	59.5	26	14.5
Hotlines	54	32	14
Daily affirmations	51	31.5	17.5
Quizzes	41	31	28
Games	40.5	30	29.5
*For you personally, how important would it be for the app/mobile website to be very confidential*?	**Very important**	**Somewhat important**	**Not at all important**
	64.5	23	12.5
**Usefulness and appeal**	**Percent of Sample (%n)**		
*Level of agreement with each statement*	**Strongly Agree or Agree**	**Neutral**	**Disagree or Strongly Disagree**
This program seems like it would be helpful for young men living with HIV	75 / 20	3.5	1 / 0.5
I would be able to use this program	65.5 / 29	4	1 / 0.5
This program is appealing to me	52 / 39.5	6	1.5 / 1
*How helpful do you think this app would be …*	**Very helpful**	**Somewhat helpful**	**Not at all helpful**
For people who were recently diagnosed with HIV?	90.5	8.5	1
For people who have had HIV for 2 years or longer?	76	22.5	1.5
*We are considering making the program an app, a mobile website, or both. What would you most likely use*?	**Percent**		
Both	53.5	–-	–-
App	41.5	–-	–-
Mobile website	4	–-	–-
Other	1	–-	–-
*About how often do you think you would use the program app or mobile website*?	**Percent**		
Several times a day	21	–-	–-
About once a day	14.5	–-	–-
About 3–4 times per week	22.5	–-	–-
About 1–2 times per week	24.5	–-	–-
About once a month	4	–-	–-
About 2- 3 times per month	13.5	–-	–-
Never	0	–-	–-

^a^Select all that apply/mutually inclusive

A large majority of respondents agreed that the program concept was appealing (91.5%) and that it would be “very helpful” for both people recently diagnosed with HIV (90.5%) and those who have had HIV for two or more years (76%). Most respondents said the program should include both a mobile app and a website (53.5%), followed by an app only (41.5%) or mobile website only/other (5%). All participants said that they would use the program. When asked how often they thought they would use the program, 35.5% of respondents said they would use it at least once a day, and 82.5% said they would use it at least once a week.

Respondents reported the proposed tools and resources would be useful and helpful, in particular the CD4/viral load tracker and adherence tool (81.5% and 75%, respectively, would “definitely” use these). Over 60% said they would “definitely” use a series of proposed community features (e.g., message forum, virtual support groups, and direct messages). Respondents indicated that the following would motivate them to continue using the app: interacting with other people living with HIV in the app community (83.5%); regularly adding new videos of people living with HIV telling their stories (73.5%) and of experts (72.5%); and the resources (73%) and tools (65.5%) on the app.

#### Usability testing of the functional prototype

Most usability testing participants completed their tasks on the working web-based prototype quickly, with many completing them within a few seconds (see Table 6 for average task completion times), indicating that the program prototype was intuitive and easy to navigate. The prototype scored a 95.7 out of 100 on the SUS, indicating very high usability [58]. When presented with lists of tools (e.g., medication reminder, CD4/viral load tracker, and provider messaging), resources (e.g., mental health and support, housing, substance use treatment, and job and career), and community features (e.g., virtual support groups, ability to message mentors and other users, and ability to post videos or photos) that the program would include, participants shared that the content and features included the most important things that a person living with HIV would need. All participants said that they had never seen a website or app with our proposed program’s comprehensive content and tools. Nearly all favored having program content that is mostly video-based versus text-based. All participants said they would use the program, though with varying frequency—some would use it daily and others weekly or monthly.

**Table 6:** Mean and range of time for each task, in seconds

	**Mean**	**Min**	**Max**
**Finding information or videos about the following:**			
Disclosing HIV status to sex partners	29.0	5	62
Disclosing your HIV status to family	16.3	7	30
Others sharing their experiences around their HIV diagnosis	15.7	2	90
Others talking about what makes them happy	21.2	8	42
Someone talking about working with his doctor	19.1	4	72
Taking HIV medication	19.4	3	60
Dealing with stress	13.3	2	40
**Finding or using the following resources or tools:**			
Help accessing housing	10.8	2	35
Help accessing support groups	29.1	4	100
The viral load/CD4 tracker	9.8	2	43
The medication reminder	8.0	3	16
Profiles of other users of the app	14.7	2	35

Twenty-one participants total completed six tasks each

## Discussion

**Fig. 1:**
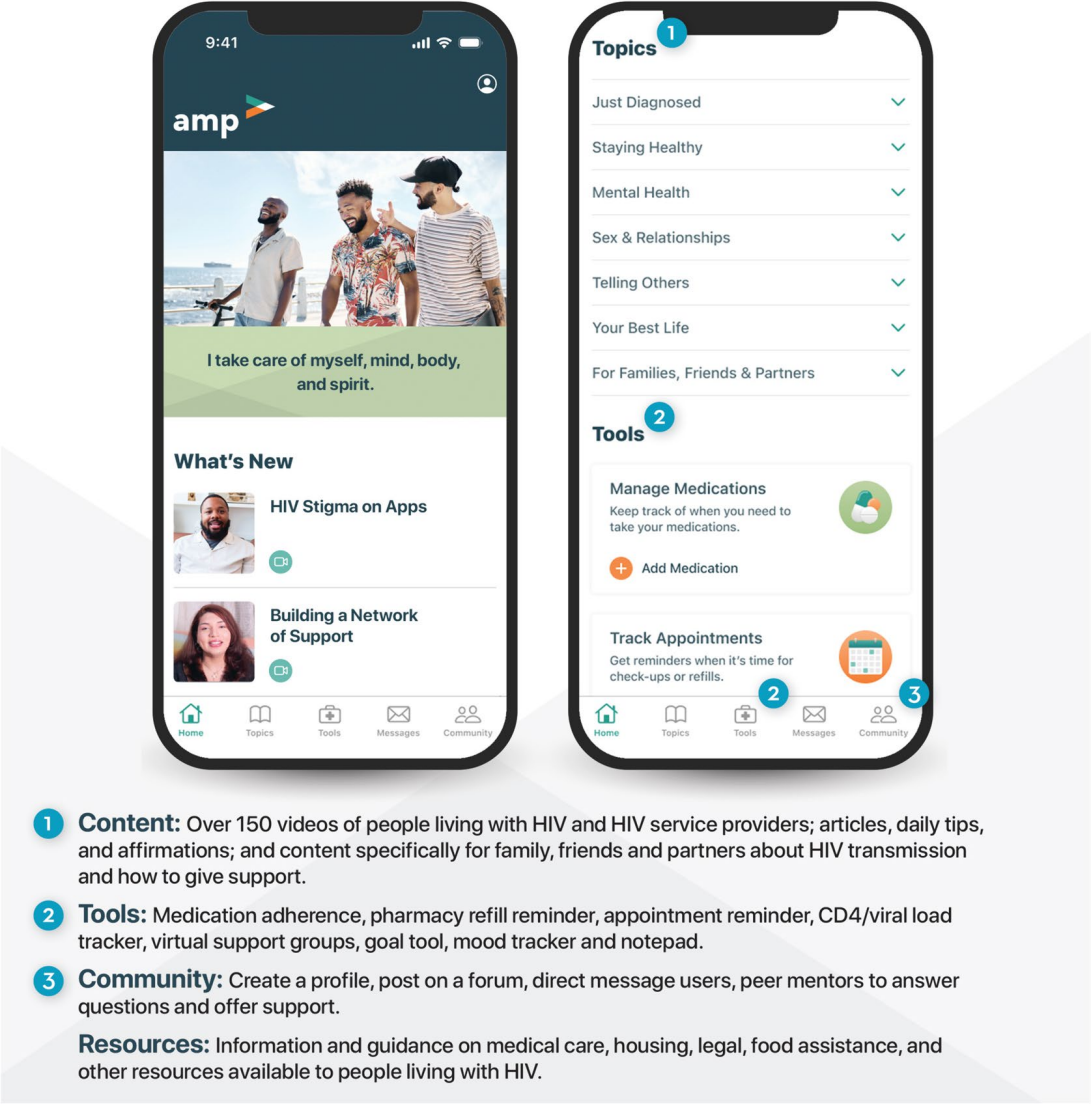
Final Amp user interface, content topics, tools, and features

This study details the use of HCD to create Amp (Fig. 1), a holistic mobile app created to improve the health and well-being of YBGBM living with HIV. Insights gathered across three stages of research—ideation, rapid prototyping, and user feedback—were iteratively incorporated to develop a program prototype that received high acceptability and usability ratings. HCD is a comprehensive approach that has been increasingly utilized in the development of mHealth programs [54, 59]. As demonstrated in this study, HCD is an approach that can yield high usability, feasibility, acceptability, and potential uptake from end users. The use of HCD in this study, which included engaging YBGBM living with HIV and HIV service providers as co-creators, resulted in a desirable program with a combination of content, tools, and resources that participants had not previously seen all in the same place. Further, the Amp concept was described by providers as promising for engaging YBGBM living with HIV across the care continuum, which is imperative to reducing HIV-related transmission, morbidity, and mortality [4, 5, 7].

Findings from our HCD research reinforced those identified in prior research regarding the needs of people living with HIV, including: social support [60], adherence support [53], HIV knowledge [61], programs to address stigma and discrimination [6, 11, 25, 62], and the general demand for more holistic approaches to HIV care [30, 31, 53]. Amp includes content, tools, and features to address all of these needs. Amp offers several alternatives to the ways that YBGBM living with HIV usually receive support—including community features that allow users to create a profile, communicate with peers via forums and direct messaging, professionally produced video narratives, trained mentors who can be messaged on-demand, and a range of tools to support medication adherence, retention in care, and viral suppression. This wide variety of tools and resources is designed to meet the needs of YBGBM where they are, and as their needs change over time. Further, HIV service providers were very enthusiastic about using Amp within their organization, to improve their services and help clients reach their HIV care continuum goals. While several existing mHealth HIV care continuum interventions take a comprehensive approach [63], Amp is unique with regard to the holistic range of tools and content, and in its extensive use of custom-created personal narrative video content.

Our study has strengths stemming from the mixed methods approach it utilized. Intensive involvement of program end users and stakeholders is fundamental to designing interventions using HCD. During every stage of our research, we collected data from geographically diverse groups of YBGBM and providers, validating and triangulating findings at each stage. This is likely the reason that Amp was so well received in the final user feedback stage.

While this study outlines a design process that was effective, there are several limitations to our findings that should be noted. Recruiting a sample of 200 HIV positive YBGBM for participation in a survey is logistically challenging, and it was not possible to ensure that this sample is representative of the broader population. Thus, our survey findings may not be generalizable to all YBGBM living with HIV in the U.S. Similarly, relying on our professional network of HIV service providers for much of the recruitment may have introduced some bias, especially as this meant that most YBGBM participants were linked to care. We attempted to mitigate this potential bias by working with multiple HIV service providers from diverse organizations who had direct connections to local populations, as well as by recruiting from a geographically diverse sample of YBGBM in stage 3, mostly via geospatial apps. Survey findings mirrored findings from other stages, which suggests that responses from stages 1 and 2 were minimally biased by our recruitment methods. Further, we administered the SUS verbally with usability testing participants over Zoom, which is different from the typical questionnaire-based approach. While we requested that participants give honest feedback in order to facilitate improvements to the app, it is possible that this approach resulted in response bias.

The results of these activities have led to the production of Amp, a mobile app that has the content, tools, community, and resources identified as important among YBGBM and HIV service providers in our HCD process. At the time of writing, the Amp mobile app is being developed with Phase II funding through a Small Business Innovation Research Grant provided by the National Institutes on Minority Health and Health Disparities. Amp will be evaluated in a nationwide randomized controlled trial in 2024, which will test the effectiveness of the application on knowledge, social support, linkage to care, medication adherence, retention in care, viral suppression, mental health, and HIV related stigma among a relatively large sample of YBGBM living with HIV. Our team plans to disseminate Amp to organizations nationwide, including HIV service organizations, public health departments, and community organizations. Ideally, Amp will be offered to people immediately after an HIV diagnosis as well as to existing clients who are living with HIV.

This study adds to the limited research on using HCD to develop HIV mHealth programs. The evaluation study will be among the first randomized controlled studies of an HIV intervention developed with HCD. Although we attribute the early signs that Amp will be feasible, acceptable, usable, and effective to the HCD process through which it was developed, further research is needed to understand the impact of HCD relative to other approaches. Future studies could also investigate using HCD to create mHealth self-management programs for other chronic illnesses.

## Conclusions

We used HCD in a highly collaborative, multi-stage process to develop an innovative mobile app that holistically addresses multiple health and wellness needs identified by YBGBM and HIV service providers. Each stage of the process identified barriers and facilitators to program uptake, with findings used to make the app more useful and engaging. These results support prior studies finding that intensive involvement of end users and stakeholders using HCD can yield innovative, acceptable, and culturally-appropriate programs.

## Data Availability

Fully de-identified data can be made available from the corresponding author on reasonable request (EMAIL ADDRESS BLINDED FOR REVIEW).

## Abbreviations

YBGBM: Young Black gay and bisexual men
MSM: Men who have sex with men
HCD: Human-centered design
CD4: CD4 T lymphocytes; CD4 cells or T-cells
SUS: System usability scale
